# Independent Action between DvSnf7 RNA and Cry3Bb1 Protein in Southern Corn Rootworm, *Diabrotica undecimpunctata howardi* and Colorado Potato Beetle, *Leptinotarsa decemlineata*

**DOI:** 10.1371/journal.pone.0118622

**Published:** 2015-03-03

**Authors:** Steven L. Levine, Jianguo Tan, Geoffrey M. Mueller, Pamela M. Bachman, Peter D. Jensen, Joshua P. Uffman

**Affiliations:** Regulatory Sciences, Monsanto Company, St. Louis, Missouri, United States of America; Kansas State University, UNITED STATES

## Abstract

In recent years, corn rootworm (CRW)-resistant maize events producing two or more CRW-active Bt proteins have been commercialized to enhance efficacy against the target pest(s) by providing multiple modes of action (MoA). The maize hybrid MON 87411 has been developed that produces the CRW-active Cry3Bb1 *Bt* protein (hereafter Cry3Bb1) and expresses a RNAi-mediated MoA that also targets CRW. As part of an environmental risk assessment for MON 87411, the potential for an interaction between the CRW-active DvSnf7 RNA (hereafter DvSnf7) and Cry3Bb1 was assessed in 12-day diet incorporation bioassays with the southern corn rootworm (SCR, *Diabrotica undecimpunctata howardi*). The potential for an interaction between DvSnf7 and Cry3Bb1 was evaluated with two established experimental approaches. The first approach evaluated each substance alone and in combination over three different response levels. For all three response levels, observed responses were shown to be additive and not significantly different from predicted responses under the assumption of independent action. The second approach evaluated the potential for a fixed sub-lethal concentration of Cry3Bb1 to decrease the median lethal concentration (LC_50_) of DvSnf7 and vice-versa. With this approach, the LC50 value of DvSnf7 was not altered by a sub-lethal concentration of Cry3Bb1 and vice-versa. In addition, the potential for an interaction between the Cry3Bb1 and DvSnf7 was tested with Colorado potato beetle (CPB, *Leptinotarsa decemlineata*), which is sensitive to Cry3Bb1 but not DvSnf7. CPB assays also demonstrated that DvSnf7 does not alter the activity of Cry3Bb1. The results from this study provide multiple lines of evidence that DvSnf7 and Cry3Bb1 produced in MON 87411 have independent action.

## Introduction

Genetically engineered (GE) insect-resistant crops that produce two or more insect-resistant traits either through co-expression of both traits in a single event or through conventional breeding have become progressively more common in commercial cultivation. In the 41 commercial insect-resistant GE maize events listed in ISLI-CERA GM crop database, 19 events express two or more insecticidal Bt proteins [1]. In the United States and many other countries, regulatory approval for import or cultivation of GE crops with combined insect-resistant traits may require developing data to demonstrate the lack of a synergistic interaction between the plant incorporated protectants (PIPs) that would impact the safety assessment [2, 3]. Safety assessments for combined insect-resistant traits can be performed by either testing the PIPs in combination or separately. Demonstrating independent action between PIPs provides convincing evidence that no unexpected adverse effects on non-target organisms (NTOs) are anticipated as a result of combining two or more PIPs and allows for the traits to be evaluated independently. This approach has become a well established procedure of safety assessments for combined trait products by many regulatory authorities [2, 3]. In 2009, the FIFRA SAP (Federal Insecticide, Fungicide and Rodenticide Act Scientific Advisory Panel) recommended for new combinations of PIPs that have been previously registered as individual events with a proven safety record, that a synergy less than ten-fold will not require additional NTO testing [3]. The rationale for this recommendation may reflect the need to achieve a safety factor of ≥10 to 100 times the expected environmental concentration, which is the margin of safety generally required for Tier I NTO assessments. However, for combinations of PIPs with different modes of action (MoAs) or novel PIPs, the SAP recommended a requirement of additional NTO testing when synergism levels are as low as five-fold [3]. It is unclear why this recommendation differed for PIPs with different or novel modes of action and may reflect a lack of a proven safety record.

Environmental safety assessments for the Cry3Bb1 protein (hereafter Cry3Bb1) producing maize were initially performed over a decade ago to support the registration of first (MON 863) and second (MON 88107) generation corn rootworm (CRW, *Diabrotica* spp.) protected maize products [4]. These safety assessments demonstrated that cultivation of the Cry3Bb1 producing maize hybrids will not result in adverse effects to NTOs including threatened and endangered species [4, 5]. In recent years, CRW-resistant Bt-maize hybrids expressing two or more CRW-active Bt proteins have been commercialized to enhance efficacy and to mitigate potential insect resistance by providing multiple MoAs against target pest species [6–9]. Cry3Bb1 was shown not to interact with the CRW active Cry34/35Ab1 binary protein in a sensitive insect bioassay [10]. The original Cry3Bb1 CRW-protected maize hybrids have also been combined with other Bt PIPs that provide control of targeted lepidopteran species [11–13]. Studies with sensitive insect species demonstrated no interaction between Cry3Bb1 and the lepidopteran-active Bt proteins including Cry1Ab protein, a combination of Cry1A.105 and Cry2Ab2 proteins, and a combination of Cry1A.105, Cry2Ab2, and Cry1F proteins [4, 10, 12].

Most recently, Monsanto Company has developed MON 87411 that confers protection against CRW and tolerance to the herbicide glyphosate. MON 87411 contains a suppression cassette that expresses an inverted repeat sequence designed to match the sequence of western corn rootworm (WCR; *Diabrotica virgifera virgifera*). The expression of the suppression cassette results in the formation of a double-stranded RNA (dsRNA) transcript containing a 240 bp fragment of the WCR Snf7 gene (*DvSnf7*). Upon consumption, the plant produced dsRNA in MON 87411 is recognized by the CRW’s RNA interference (RNAi) machinery resulting in down regulation of the targeted *DvSnf7* gene leading to CRW mortality [14–16]. The spectrum of activity for DvSnf7 RNA (hereafter DvSnf7) produced in MON 87411 is narrow and activity is only evident in a subset of beetles within the Galerucinae subfamily of Chrysomelidae within the order Coleoptera [17]. The high specificity of DvSnf7 greatly reduces the likelihood of adverse effects on NTOs, including those beneficial to agriculture. MON 87411 also contains a *cry3Bb1* gene that produces a modified *Bacillus thuringiensis* (subsp. *kumamotoensis*) Cry3Bb1 to protect against CRW larval feeding. Cry3Bb1 expressed in MON 87411 has been commercialized since 2003 in transgenic Bt-maize hybrids and offers an additional means of control against corn rootworms [4, 18, 19]. Presently, the scientific community has a comprehensive understanding of the MoA of Bt proteins [20]. Cadherin-like proteins are the receptors for Cry Bt proteins including the coleopteran-active Cry Bt toxins [21–23] and the binding to cadherin proteins results in insect death by activation of an oncotic cell death pathway and/or formation of toxin oligomers that result in pore formation and osmotic cell shock [24–26]. The insecticidal activity of Cry3Bb1 has also been shown to be only evident with certain species of the coleopteran Chrysomelidae family, particularly the CRW complex *Diabrotica* spp. [7, 19, 27].

The objective of this study was to investigate the potential for an interaction between DvSnf7 and Cry3Bb1 in a sensitive insect species and to interpret the results in the context of an environmental risk assessment for MON 87411 maize. The potential for an interaction between DvSnf7 and Cry3Bb1 was evaluated with two established experimental approaches. The first approach assessed additivity by evaluating the response of each substance alone and in combination at approximately equipotent concentrations. Three different mixtures with increasing effective concentrations of DvSnf7 and Cry3Bb1 were tested in 12-day southern corn rootworm (SCR, *Diabrotica undecimpunctata howardi*) growth inhibition assays. The second approach evaluated the potential of a fixed sub-lethal concentration of Cry3Bb1 to decrease the 12-day median lethal concentration (LC_50_) of DvSnf7 and *vice versa*. The later approach has been recommended as an alternative design to assess for a potential interaction [28, 29] and is frequently used in interaction studies with mixtures between Bt proteins [30–32] and between synthetic chemicals [33, 34]. A third set of bioassays to further evaluate the potential for an interaction was performed with mixtures containing a fixed concentration of DvSnf7 and an effective concentration of Cry3Bb1 against the Colorado potato beetle (CPB, *Leptinotarsa decemlineata*). CPB was selected as an additional test species because it is sensitive to oral dsRNA and Cry3Bb1 but DvSnf7 has no biological activity against CPB because of lack of sufficient sequence identity [17, 35, 36].

## Materials and Methods

### Test materials

The 968 nucleotide (nt) DvSnf7 used in this study was synthesized *in vitro* with T7 RNA polymerase. DvSnf7 was suspended in ultrapure distilled water (Life Technologies, Grand Island, NY). The DvSnf7 concentration was determined to be 2.21 mg/ml using NanoDrop 8000 (Thermo Scientific, Wilmington, DE). Aliquots of the DvSnf7 sample were stored in a −80°C freezer when not in use.

Cry3Bb1 used in this study was expressed and purified from *E*. *coli*. The concentration of Cry3Bb1 was determined by amino acid compositional analysis to be 10.19 mg/ml and a purity of 97%. Biological activity was confirmed by diet incorporation bioassay against CPB with a 7-day LC_50_ of 0.60 μg/ml diet, which is in the LC_50_ range of reported activity for CPB in a comparable 7-day assay [35]. Aliquots of the Cry3Bb1 sample were stored in a −80°C freezer when not in use.

### Insects

SCR eggs purchased from Crop Characteristics, Inc. Farmington, MN were incubated at target temperatures ranging from approximately 10°C to 27°C to obtain the desired hatch time. Newly-hatched SCR larvae (≤30 hours after the first observation of hatching) were used in all SCR bioassays. SCR has been used in our laboratory as a surrogate for the WCR due to its relative ease of laboratory rearing and handling [37]. Additionally, SCR are highly sensitive to DvSnf7 [14, 17] and Cry3Bb1 [27]. CPB eggs were purchased from French Agriculture Research, Inc. (Lamberton, MN) and were held at a target temperature of 15°C prior to being washed and incubated for hatching at a target temperature of 27°C to obtain the desired hatch time. Newly-hatched CPB larvae (≤30 hours after the first observation of hatching) were used in all CPB bioassays.

### SCR diet incorporation bioassay

The biological activity of DvSnf7 and Cry3Bb1 against SCR were assessed in diet incorporation bioassays. A DvSnf7 stock solution was prepared in ultrapure distilled water (Life Technologies, Grand Island, NY) and the Cry3Bb1 stock solution was prepared in a buffer solution containing 10 mM sodium carbonate/bicarbonate, 0.1 mM EDTA at pH 10. Dosing solutions were prepared in 2 ml of purified water across a series of 7 or 9 concentrations with a 2-fold separation factor between concentration levels. Treatment diets were then prepared by mixing the 2 ml dosing solution with 8 ml warm (48–56°C) agar-based SCR diets (Bioserv, Frenchtown, NJ). Diets were vortex-mixed until homogeneous and dispensed in 0.25 ml aliquots into 48-well plates (Falcon, Corning, NY). Each well was targeted to be infested with a single SCR larva and each treatment contained 30 to 42 insects. Finally, each plate was covered with mylar and a small hole was made in each well for ventilation. Bioassays were incubated in an environmental chamber programmed at 27°C, 70% relative humidity and in total darkness.

To assess growth inhibition (GI), the number of surviving or dead larvae and the combined body mass of surviving larvae were recorded for each concentration level at the end of the 12-day test period. SCR was assumed dead if it did not respond after probing. A control diet containing the same volume of ultrapure distilled water (hereafter water control) was included to correct background mortality and to calculate GI as a percent of control for bioassays with DvSnf7. A control diet containing the same volume of buffer solution as in the highest Cry3Bb1 concentration level (hereafter buffer control) was also included to correct for background mortality and to calculate GI as a percent of control in bioassays with Cry3Bb1. The mortality or average body mass of the surviving insects from the pooled water and buffer control was used to correct for background mortality or GI as a percent of pooled controls in bioassays with mixtures of Cry3Bb1 and DvSnf7. GI was calculated as percent reduction in average body mass of surviving insects in each treatment in relation to the appropriate control.

### Time-course for SCR mortality and growth inhibition for DvSnf7 and Cry3Bb1

**Fig 1:**
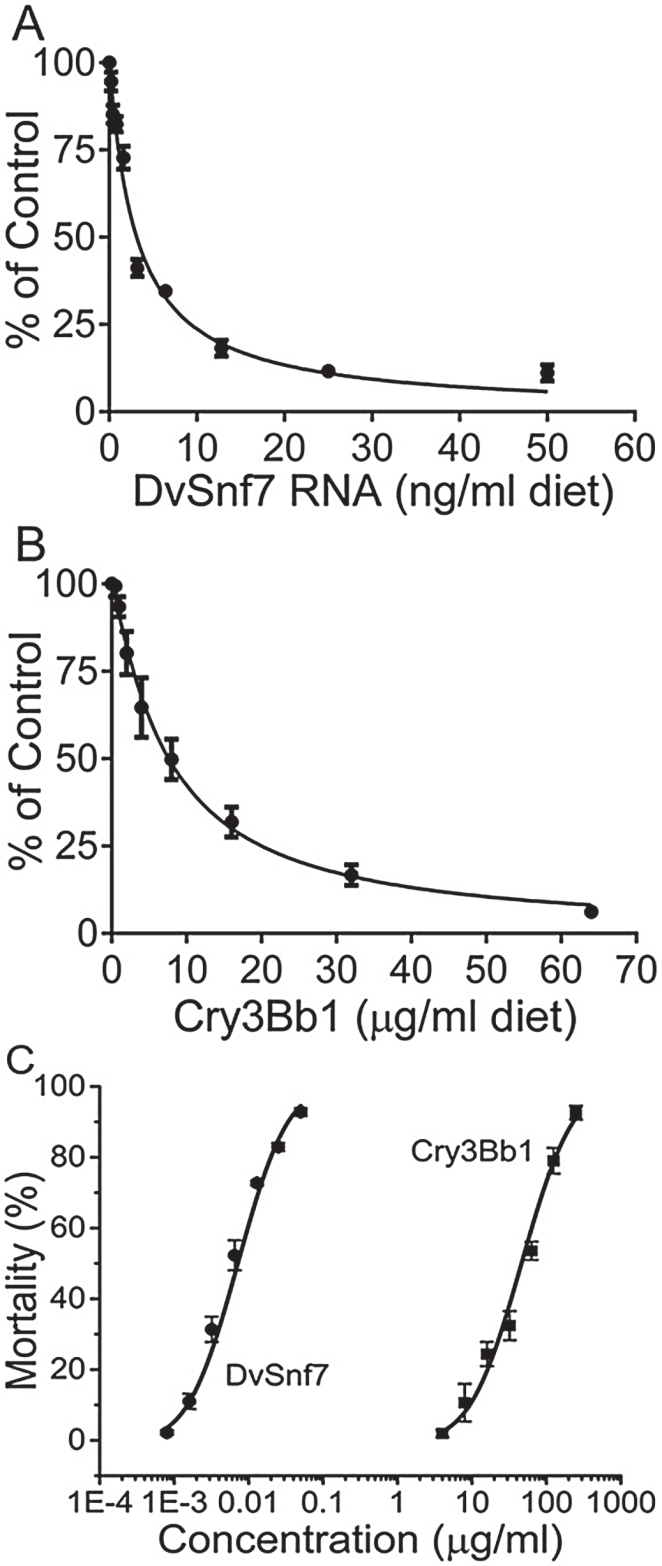
Concentration response curves for growth inhibition activity of DvSnf7 (A) and Cry3Bb1 (B) and for mortality of DvSnf7 and Cry3Bb1 (C) to SCR in 12-day diet incorporation bioassays. Data points represent mean values with standard errors of six replicated bioassays for growth inhibition curves (A and B) and of five replicated bioassays for mortality curves (C). The concentration-mortality response curves were plotted with log transformation of concentrations (X-abscissa) plotted against the percent mortality (Y-abscissa).

For bioassays to compare the time-course for growth inhibition of DvSnf7 and Cry3Bb1, each substance was tested at a single concentration level that results in 80% growth inhibition (GI_80_). The GI_80_ values were estimated from concentration response curves performed in preliminary bioassays (Fig. 1A and B) and were tested at 25 μg Cry3Bb1/ml diet and 0.012 μg DvSnf7/ml diet. Each treatment included 15 plates and each plate had 42 individually housed SCRs. Water and buffer controls were included with the same number of plates and same number of SCR for each plate. The number of survivors or deaths and the combined body mass of surviving insects was recorded every day from one plate for each treatment from days 1 to 10 and from two plates of each treatment at days 11 and 12. GI was calculated based on the reduction in mean body mass of surviving SCR in the treatment of Cry3Bb1 or DvSnf7 in relation to the buffer control or water control, respectively.

To assess the time-course for mortality, DvSnf7 and Cry3Bb1 were tested at concentrations of 250 μg Cry3Bb1/ml or 0.05 μg DvSnf7/ml diet, which approximates the LC_95_ values based on concentration response curves from preliminary bioassays (Fig. 1C). Mortality was recorded daily from the same plate with 36 individually housed SCRs. Water and buffer controls were included to correct for background mortality in DvSnf7 and Cry3Bb1 treatments, respectively.

### Combined effects assessment for DvSnf7 and Cry3Bb1 in equipotent concentrations

To evaluate the combined effect for DvSnf7 and Cry3Bb1, bioassays were conducted with mixtures containing each substance at approximately equipotent concentrations. Concentration levels for DvSnf7 and Cry3Bb1 were selected from concentration response profiles in preliminary bioassays (Fig. 1A and B) and were tested at 0.0015 μg DvSnf7/ml diet and 4.0 μg Cry3Bb1/ml diet, 0.003 μg DvSnf7/ml diet and 8.0 μg Cry3Bb1/ml diet, 0.006 μg DvSnf7/ml diet and 16.0 μg Cry3Bb1/ml diet, representing approximately 35% (low response), 50% (medium response), and 65% (high response) growth inhibition for the individual treatments. Additionally, three mixtures containing DvSnf7 and Cry3Bb1 at the low, medium, and high response levels were evaluated. Tests with each individual substance and their mixtures were run in parallel and were replicated three times.

### Combined effects assessment for DvSnf7 and Cry3Bb1 with a fixed sub-lethal concentration approach

The potential for Cry3Bb1 to interact with DvSnf7 and *vice versa* was assessed with a fixed sub-lethal concentration approach. With this approach, a fixed sub-lethal concentration was used to evaluate if there would be a change in 12-day median lethal concentrations (LC_50_). The fixed sub-lethal concentrations for DvSnf7 and Cry3Bb1were estimated to be the level that produces a 10% growth inhibition (GI_10_) in 12-day bioassays (Fig. 1A and B).

Treatments in each experiment were run in parallel using the same batch of SCR and included: (1) Cry3Bb1 at 1.11 μg/ml diet, (2) DvSnf7 at 0.00035 μg/ml diet, (3 and 4) Cry3Bb1 concentration response (from 4 to 250 μg/ml diet) in the presence and absence of 0.00035 μg DvSnf7/ml diet, and (5 and 6) DvSnf7 concentration response (from 0.0008 to 0.050 μg/ml diet) in the presence and absence of 1.11 μg Cry3Bb1/ml diet. In addition, three water and buffer controls were included in each experiment. Each treatment was replicated three times on separate days each using a different batch of SCR.

### Assessment of potential modulation of Cry3Bb1 activity against CPB in the presence of a fixed-concentration of DvSnf7

The insecticidal activity of Cry3Bb1 against CPB was evaluated in the presence and absence of a fixed-concentration of DvSnf7 of 1000 ng/ml in 12-day diet-incorporation bioassays. This concentration of DvSnf7 was chosen because it greatly exceeds the LC_50_ value for the Snf7 ortholog against CPB and this level has no effect on CPB when tested alone [17]. Treatment diets were prepared by mixing the test substance(s) in 5 ml of water and then mixed with an agar-based CPB diet (BioServ, Frenchtown, NJ) to achieve a final volume of 25 ml. All bioassays included five concentrations with a two-fold separation factor between test concentrations. Each bioassay included an assay control replicate and a buffer control replicate. Each concentration level tested a target number of 32 individually housed CPB larvae (≤30 hours from the first observation of hatch). Trays were covered with ventilated adhesive covers and incubated at a target temperature of 27°C, 60% relative humidity, and a day length of 14 hours light:10 hours dark. Bioassays were replicated three times on separate days with separate batches of CPB. At the end of the bioassay, the number of insects in each concentration level and the number of surviving insects and/or dead insects in each concentration level was recorded.

### Data Analysis

LC_50_ values were estimated by Probit analysis using a logistic distribution in SAS (version 9.2, SAS Institute Inc., Cary, NC). Growth inhibition values were estimated with a standard 3-parameter logistic regression (Equation 1) in GraphPad Prism 6 for Windows (Version 6.03).

$$Y=W0/\left(1+{\left(X/GI_{X}\right)}^{b}\right)$$ 1

Where, *Y* is the percent body mass at each concentration level in relation to the control (% of control). *W*0 is the top response value, i.e. percent of the body mass in control treatment which is normalized to 100%. *X* is the concentration level tested in each bioassay with DvSnf7 or Cry3Bb1. *GI*_*X*_ is the effective concentration for growth inhibition at level of *Y*, e.g. *G*_10_ is a concentration causing 10% growth inhibition of surviving SCRs in relation to control, and *GI*_50_ is a concentration resulting in 50% growth inhibition of surviving SCRs in relation to control. *b* is the slope factor of the regression curve.

The prediction of mixture toxicity was calculated with the response addition (RA) model (Equation 2). Response addition can be used to evaluate mixtures when substances have different modes of action [38, 39].

$$E\left(c_{MIX}\right)=E\left(c_{A}\right)+E\left(c_{B}\right)-\left[E\left(c_{A}\right)\times E\left(c_{B}\right)\right]$$ 2

Where *E*(c_*MIX*_) is the prediction of total effect of the mixture, E(c_*A*_) and E(c_*B*_) are the observed effect caused by individual Cry3Bb1 and DvSnf7, respectively. Predicted and observed values were compared with a t–test (α = 0.05). When the observed mixture effect is not significantly different from the predicted mixture effect, the combined action is then considered to be independent without interaction [40–42].

## Results and Discussion

### Biological activity of DvSnf7 and Cry3Bb1 against SCR

DvSnf7 and Cry3Bb1 demonstrated a concentration-dependent relationship against SCR for both lethality and growth inhibition (Fig. 1). The estimated concentrations that resulted in 50% growth inhibition (GI_50_) of DvSnf7 and Cry3Bb1 were estimated to be 0.0031 μg/ml diet (95% CIs: 0.0028–0.0035) and 7.6 μg/ml diet (95% CIs: 6.3–8.9), respectively. The shape of both concentration-response curves was comparable with similar slopes of 1.0 ± 0.1 for DvSnf7 and 1.1 ± 0.1 for Cry3Bb1. The estimated concentration that resulted in 10% growth inhibition (GI_10_) was estimated to be 0.00035 μg/ml diet for DvSnf7 and 1.11 μg/ml diet for Cry3Bb1 and these GI_10_ values were used as the fixed sub-lethal concentrations in the assessments of combined effects following an approach outlined by Tabashinik and Pöch et al. [28, 29]. The GI_80_ concentrations were estimated to be 0.012 and 25 μg/ml diet for DvSnf7 and Cry3Bb1, respectively, and these estimated concentrations were used to assess time-dependent SCR growth inhibition for DvSnf7 and Cry3Bb1. The GI_35_, GI_50,_ and GI_65_ concentrations were estimated from the concentration responses (Fig. 1 A and B) and were then used for the assessment of the combined effects of DvSnf7 and Cry3Bb1.

Concentration-mortality responses for DvSnf7 and Cry3Bb1 were fully characterized in five replicated diet-incorporation bioassays (Fig. 1C). LC_50_ values for DvSnf7 and Cry3Bb1 were estimated to be 0.0071 μg/ml diet (95% CIs: 0.0041 to 0.012) and 46 μg/ml (CIs: 27 to 79), respectively. The concentration-mortality response curves had a similar slope, 1.8 ± 0.1 for DvSnf7 and 1.9 ± 0.1 for Cry3Bb1. DvSnf7 and Cry3Bb1 concentrations that resulted in approximately 95% mortality (LC_95_) were used to characterize the time-dependent mortality profile for DvSnf7 and Cry3Bb1.

### Differences in time to effect for DvSnf7 and Cry3Bb1

**Fig 2:**
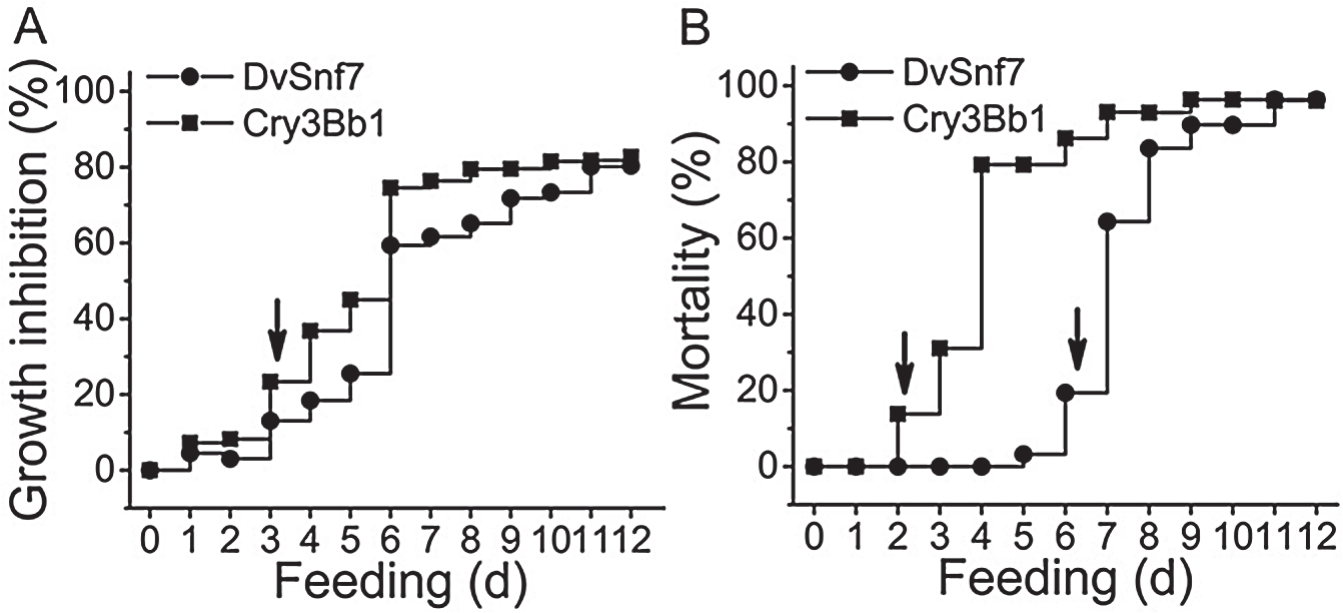
Time-to-effect relationships for SCR growth inhibition (A) and mortality (B) of DvSnf7 (●) and Cry3Bb1 (■) in 12-day diet incorporation bioassays. Arrows indicate the onset of significant growth inhibition or mortality. The time-to-effect for growth inhibition was assessed at a concentration of 0.012 μg DvSnf7/ml diet and 25 μg Cry3Bb1/ml diet which approximated the GI_80_ concentration. Each treatment included 15 plates and each plate had 42 individually housed SCR larvae. Water and buffer controls were included with the same number of plates and same number of SCR for each plate. Growth inhibition was calculated based on the reduction in mean body mass of surviving SCR in relation to the appropriate control. The time-to-effect for mortality was assessed at concentrations of 250 μg Cry3Bb1/ml diet and 0.050 μg DvSnf7/ml diet which approximated the LC_95_ concentration. Mortality was recorded daily from the same plate with 36 individually housed SCR larvae and was corrected for background mortality.

**Fig 3:**
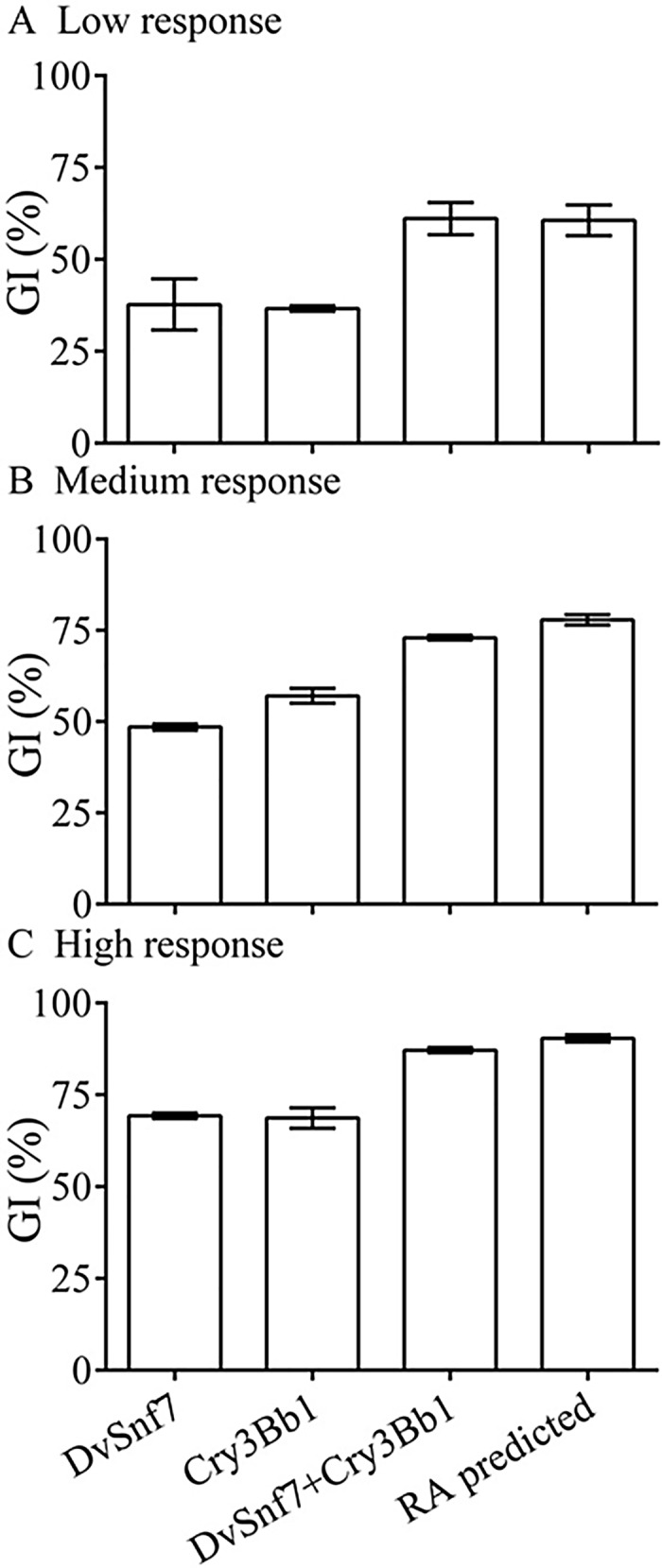
Observed growth inhibition (GI) with standard errors (SEs) of Cry3Bb1 and DvSnf7 and their mixtures at levels of low response (A), medium response (B) and high response (C) in 12-days diet incorporation bioassays. The predicted GI of the mixture in each level was calculated based on the GIs of two individuals using the RA model. The average GI and associated SEs were calculated from three replicates.

Experimental methods developed to evaluate the combined effects of Cry3Bb1 and DvSnf7 were tailored to account for their different modes of action [3]. Consequently, to inform the design of the interaction studies, bioassays were conducted to compare the time-to-effect relationship for SCR growth inhibition and mortality between DvSnf7 and Cry3Bb1. A single concentration that approximated the 12-day GI_80_ level was used to characterize the time-to-effect for growth inhibition and a concentration that approximated the 12-day LC_95_ level was used to characterize the time-to-effect for mortality. DvSnf7 and Cry3Bb1 had comparable time-to-effect profiles for SCR growth inhibition (Fig. 2A). Growth inhibition was evident after 3 days of feeding and reached approximately 80% for both substances by the end of the 12-day bioassay. In contrast to the growth inhibition results, DvSnf7 and Cry3Bb1 showed a very different time-to-effect pattern for SCR mortality (Fig. 2B). Significant mortality was evident by Day 2 for Cry3Bb1. However, mortality was not evident until Day 6 for DvSnf7 and was similar to the time-to-mortality pattern reported for WCR [14]. Previously, it was shown that *DvSnf7* gene expression at mRNA levels was significantly suppressed in WCR midgut and carcass as early as one day after feeding and the suppression became more pronounced by the third day post-feeding. However, DvSNF7 protein levels were significantly reduced in larvae after 5 days of feeding [14]. Because of the significant difference in the time-to-mortality effect between DvSnf7 and Cry3Bb1, mortality as the measurement endpoint could confound the assessment of combined activity. In other words, the rapid time-to-mortality of Cry3Bb1 could have created significant overt toxicity and consequently impeded the DvSnf7 effect. Therefore, growth inhibition was selected as the measurement endpoint, rather than mortality, to measure the combined action of Cry3Bb1 and DvSnf7 with equipotent concentrations (Fig. 3).

### Combined activity of DvSnf7 and Cry3Bb1 is additive

The potential for an interaction between the CRW-active DvSnf7 and Cry3Bb1 was assessed in diet bioassays with the SCR, a sensitive species to both PIPs [14, 17, 27]. The combined effect of DvSnf7 and Cry3Bb1 was evaluated over three response levels. DvSnf7 and Cry3Bb1 were mixed at approximately equipotent GI levels that represented a low response (GI_35_), a medium response (GI_50_), and a high response (GI_65_), respectively. The combined GI activity was predicted from the individual GI activities using the RA model (equation 2). The RA model is based on the assumption that each substance in the mixture exerts its activity with different physiological, biochemical systems or molecular targets but leads to a common toxicological endpoint [43]. There was good agreement between observed and predicted GI responses across the three tested mixture levels (p>0.050) (Fig. 3). These SCR results suggest independent action that is jointly additive between the DvSnf7 and Cry3Bb1.

### No interaction between DvSnf7 and Cry3Bb1 using the fixed sub-lethal concentration approach

An alternative approach to assess the potential for interaction in binary mixtures has used a fixed sub-lethal concentration design [28, 29]. With this method, toxin A was added at a fixed sub-lethal concentration to an effective concentration(s) of toxin B. This approach has been recommended to assess potential synergistic interaction between Bt proteins [28] and between binary mixtures of synthetic chemicals [29]. A synergistic interaction can be characterized by a left shift of the location of the concentration response curve of toxin A and represents a significant decrease of the LC_50_ value by a fixed sub-lethal concentration of toxin B when toxin B alone exhibits no effect. Several studies have demonstrated the effectiveness of this approach in detecting a synergistic interaction [30–33]. For example, a trypsin inhibitor at a non-effective concentration increased the insecticidal activity of a Bt protein (Cry3A) against CPB by 8 to 40-fold [44]. Similarly, when a fixed low-level concentration of chlorpyrifos (below EC_1_ level) was added in combination with effective-level concentrations of esfenvalerate, the observed toxicity of the mixture to fathead minnows was greater than that observed for esfenvalerate only, indicating a synergistic effect of the low-level concentration of chlorpyrifos [33].

**Fig 4:**
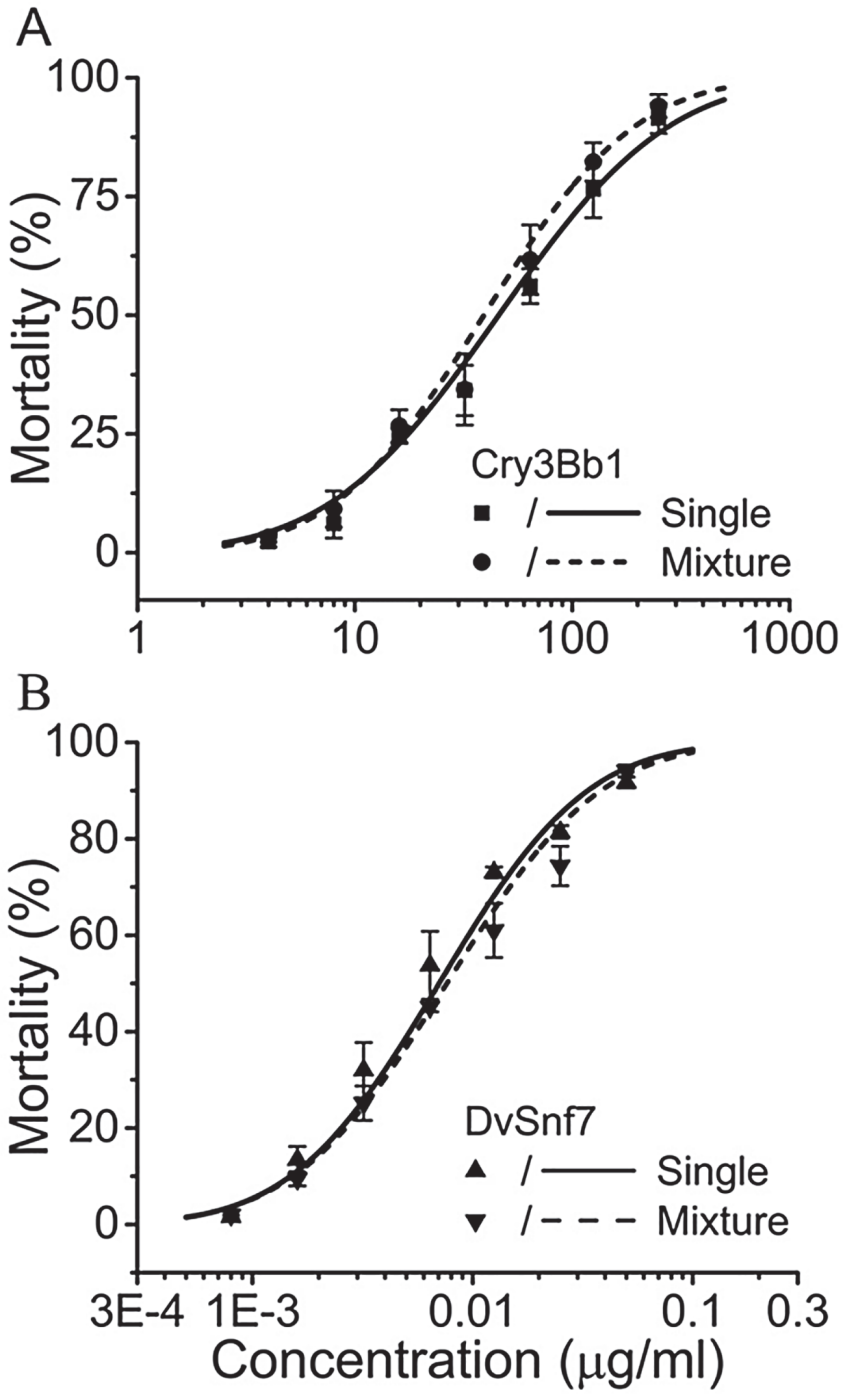
Observed concentration-mortality responses of Cry3Bb1 and DvSnf7 to the SCR. (A) Cry3Bb1 when applied alone (■) as compared to its mixture with a fixed sub-lethal concentration of DvSnf7 (●) and (B) DvSnf7 when applied alone (▲) as compared to its mixture with a fixed sub-lethal concentration of Cry3Bb1 (▲). The responses were plotted based on the tested Cry3Bb1 (A) or DvSnf7 concentrations (B), rather than the mixture concentrations. Data points represent mean mortality with standard errors of three replicated bioassays. LC_50_ values and associated 95% confidence intervals are listed in Table 1.

In the present study, the fixed sub-lethal concentration approach was used as a second confirmatory method to assess the potential for an interaction between DvSnf7 and Cry3Bb1 in 12-day diet incorporation SCR bioassays. The sub-lethal concentration is defined as a concentration inducing no apparent mortality in the experimental population [45]. The estimated GI_10_ value was selected as the sub-lethal concentration because it did not confound the SCR responses through the manifestation of overt toxicity. In bioassays with Cry3Bb1 treated at the GI_10_ concentration an average mortality of 8.1% was observed. However, this was not significantly different from the buffer control (p > 0.050), and represents only 1.4% difference in mortality after correction for the level of mortality in the buffer control. In bioassays with DvSnf7 at the sub-lethal concentration an average mortality of 4.5% was observed and this was below the level observed in the water control (5.2%). The addition of DvSnf7 at the GI_10_ concentration to increasing Cry3Bb1 concentrations resulted in a comparable concentration-mortality response curve to Cry3Bb1 alone (Fig. 4A) and the 12-day LC_50_ values of Cry3Bb1 were comparable with overlapping 95% CIs in the presence and absence of DvSnf7 (Table 1). Similarly, the addition of Cry3Bb1 at the GI_10_ concentration to increasing DvSnf7 concentrations resulted in comparable concentration-mortality response curve to DvSnf7 alone (Fig. 4B) and the 12-day LC_50_ values of DvSnf7 were comparable with overlapping 95% CIs in the presence and absence of Cry3Bb1 (Table 1). In addition, the LC_50_ values for Cry3Bb1 with and without the fixed sub-lethal DvSnf7 concentration overlapped with the LC_50_ value previously obtained for Cry3Bb1. Similarly, the LC_50_ values for DvSnf7 with and without the fixed sub-lethal Cry3Bb1 concentration overlapped with the LC_50_ value previously obtained for DvSnf7. Taken together, the comparable concentration response curves and LC_50_ values for each individual PIP and the two PIPs in combination indicate an independent action between DvSnf7 and Cry3Bb1.

**Table 1:** 12-day LC_50_ values for the Cry3Bb1, DvSnf7 and their mixtures to southern corn rootworm, *Diabrotica undecimpunctata howardi*.

Treatment	LC_50_ Value	95% Confidence intervals
Cry3Bb1	46 μg/ml	26–67
Cry3Bb1 + DvSnf7^1^	41 μg/ml	23–58
DvSnf7	6.4 ng/ml	3.5–9.2
DvSnf7 + Cry3Bb1^2^	8.2 ng/ml	4.5–12

1 DvSnf7 was added at a fixed concentration at 0.35 ng/ml diet which represents the 12-day GI_10_ concentration.

2 Cry3Bb1 was added at a fixed concentration at 1.11μg/ml diet which represents the 12-day GI_10_ concentration.

### No influence of DvSnf7 on Cry3Bb1 activity against CPB

**Fig 5:**
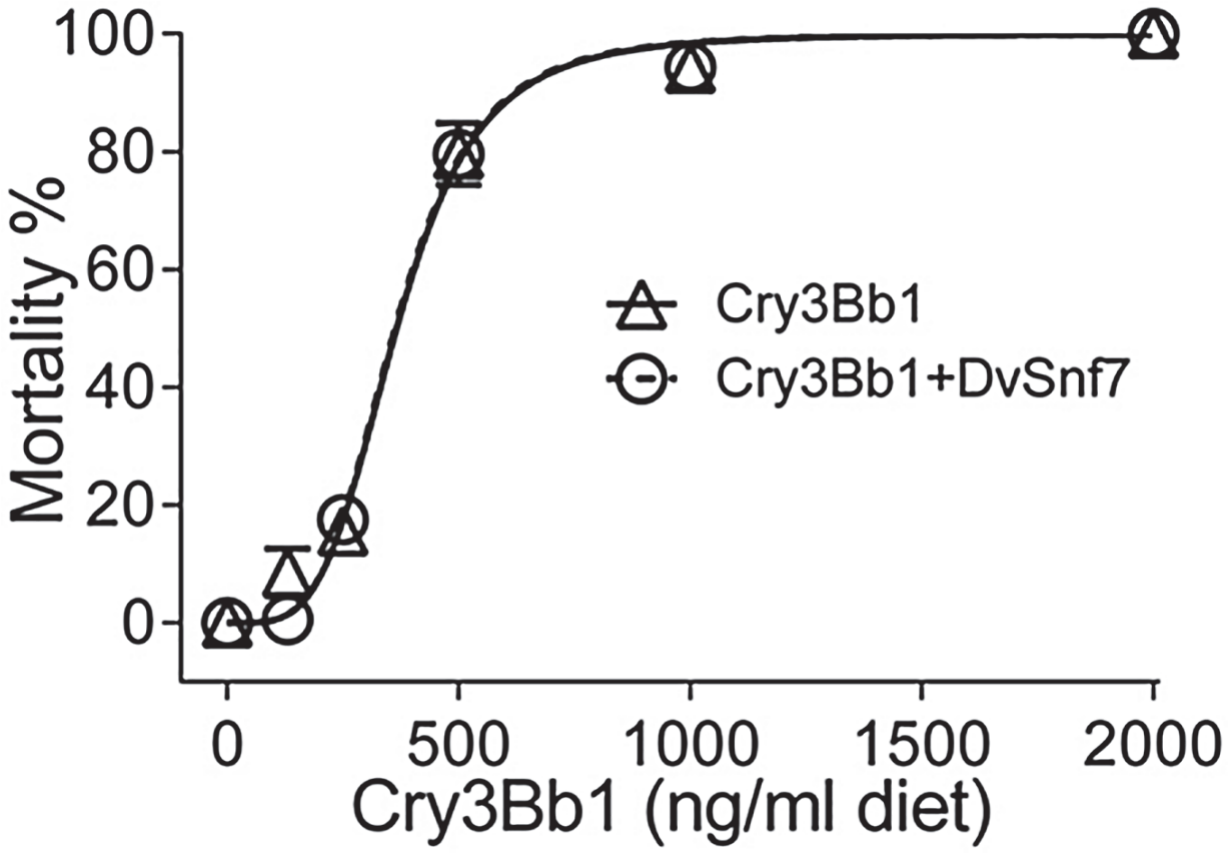
Concentration response curves of CPB mortality for Cry3Bb1 alone (Δ) and Cry3Bb1 in the presence of DvSnf7 RNA (○) at a fixed concentration of 1000 ng/ml diet in 12-day diet bioassays. The responses were plotted based on the tested Cry3Bb1 concentrations, rather than the mixture concentrations. Data points represent mean mortality with standard errors of three replicated bioassays. LC_50_ values and associated 95% confidence intervals are listed in Table 2.

In addition to the experiments with SCR that demonstrated an independent interaction between DvSnf7 and Cry3Bb1, CPB was used in an additional set of bioassays that used the fixed-concentration approach. In these assays, DvSnf7 was added at a fixed-concentration of 1,000 ng/ml diet across the range of Cry3Bb1 effective concentrations that characterized the concentration mortality relationship. As previously discussed, DvSnf7 does not have activity against CPB although CPB are susceptible to environmental dsRNA [17, 36]. The results from these assays demonstrate, as predicted, that Cry3Bb1 activity was not affected when tested in combination with DvSnf7. The 12-day LC_50_ values for Cry3Bb1 in the absence and presence of a fixed-concentration of the DvSnf7 were comparable with largely overlapping 95% CIs (Table 2, Fig. 5).

**Table 2:** 12-day LC_50_ values for the Cry3Bb1 protein and the mixture with DvSnf7 for the Colorado potato beetle, *Leptinotarsa decemlineata*.

Treatment	LC_50_ Value(ng/ml)	95% Confidence intervals(ng/ml)
Cry3Bb1	376	259–493
Cry3Bb1 + DvSnf7^*^	380	274–485

* A fixed concentration of DvSnf7 at 1000 ng/ml diet was added in each effective concentration level of Cry3Bb1 in the mixture. Concentration responses are illustrated in Fig. 5.

## Conclusion

Mixture studies with SCR and CPB collectively provide multiple lines of evidence that DvSnf7 and Cry3Bb1 produced in MON 87411 have independent action. Provided each PIP in a mixture acts independently, and there are no significant adverse effects to NTOs from either PIPs, it is possible to assess their environmental risk independently [3]. This approach has a long history of use in environmental risk assessment and satisfying the requirements of this approach enables the use of existing NTO studies performed separately for the individual PIPs to assess the safety of the combined trait product [3].
